# European Sexual Medicine Network: a unique platform for collaboration

**DOI:** 10.1038/s41443-020-0299-4

**Published:** 2020-05-14

**Authors:** Marianne Greil-Soyka, Ethel Quayle, Johannes Bitzer

**Affiliations:** 1 Chair of the European Sexual Medicine Network, Salzburg, Austria; 2 School of Health in Social Science, University of Edinburgh, Edinburgh, UK; 3 Department Obstetrics and Gynecology, University Hospitals Basel, Basel, Switzerland

**Keywords:** Preventive medicine, Translational research

## The COST Action

The European Sexual Medicine Network, “ESMN” is an international Network of medical science researchers and practitioners, educators and social service professionals involved with sexual health and medicine [1, 2]. ESMN allows its members to pool their talents, training and time in order to expand and deliver up-to-the-minute sexual health and medicine programs and services throughout the European Community and beyond. The ESMN’s fundamental focus is to improve sexual health—a state of physical, emotional, mental and social well-being in relation to sexuality—at individual, family, community and health system levels [3].

The three strategic priorities of the European Cooperation in Science and Technology, “COST” are promoting and spreading excellence, and fostering interdisciplinary research in order to further breakthrough science and empowering and retaining young researchers and innovators. The first Management Committee Meeting on April 5th, 2019 in Brussels marked the official beginning of the Network, opening doors of opportunity for medical and social scientists. Their research and practices are expanding the scope of the field. Courses and degree programs in Sexual Medicine are being designed that meet European standards for university scholars. Through the Network’s educational programs for all ages, the public is beginning to learn how, and why, good sexual health affects a person’s general health and well-being.

## An international team

The COST Action ESMN is chaired by founder, MG-S, of Salzburg, Austria. As of January 2020, the membership numbered over 100 highly committed medical professionals and social scientists [1, 2]. They come from 31 nations across Europe and beyond, including Austria, Belgium, Denmark, Finland, France, Germany, Great Britain, Greece, Italy, Netherlands, Norway, Spain, Sweden, Switzerland, Albania, Bosnia and Herzegovina, Bulgaria, Czech Republic, Croatia, Hungary, Lithuania, Latvia, Malta, Moldova, North Macedonia, Poland, Portugal, Slovenia, Serbia, Turkey, Israel and Canada.

Currently the ESMN has four working groups (WGs): WGs 1 and 2 focus on interdisciplinary research in sexual medicine, WG3 on the development of a basic curriculum for university education on sexual medicine, and WG4 on communication, evaluation and dissemination. In the Core Group—where the threads converge—14 leaders are working together internally to coordinate and disseminate the ESMN’s various programs, projects, curricula and research results out into the world and academic communities.

## Connections throughout Europe and beyond

**Fig. 1:**
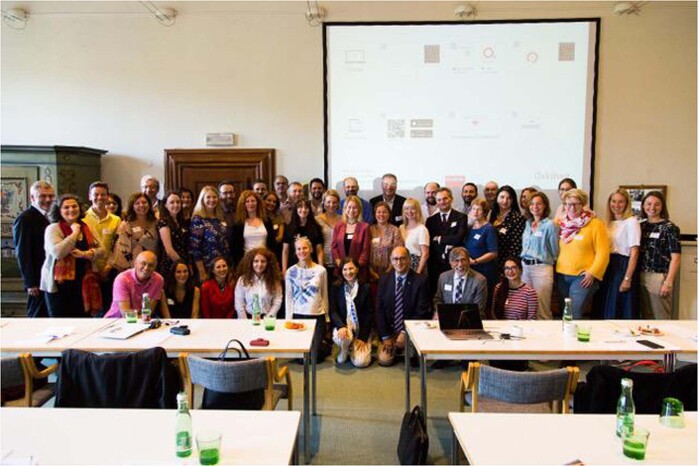
High profile launch and Management Committee (MC)-meeting of the European Sexual Medicine Network in Salzburg 2019. The meeting was held on the 14th and 15th September 2019. The picture was taken by Peter Schreiner.

Meanwhile, there have been a series of meetings initiated at the 2019 high profile launch in Salzburg (Fig. 1), followed by the Core Group Meeting and Working Group Meeting in Vienna during which the members collaborated enthusiastically to establish their group’s goals and also sketch out the timing and methods for reaching them. It seemed as though each member had been waiting for this rare collaborative opportunity the ESMN would provide. Imagine! Dozens of innovative individuals absorbed in different disciplines, different specialties such as urology, andrology, gynaecologist endocrinology, psychiatry, as well as public health disciplines, such as psychology, medical sociology and pedagogy… most of them strangers to one another, coming from different countries and cultures, with different mother tongues, meet and start working together to develop high standards. From a short and concise 15-page proposal for the COST Action, submitted by the main proposer, MG-S, this group had gathered to launch a uniquely qualified network of specialists who now could combine their multiple talents to advance sexual medicine in the European community and beyond.

## Initial results are already in sight

COST provides networking opportunities for researchers and innovators in order to strengthen Europe’s capacity to address scientific, technological and societal challenges.

A first call from the coordinator for “short-term scientific missions” was sent out and, fortunately, there has been immediate resonance. Short-term scientific missions are exchange visits between researchers involved in a COST Action, allowing scientists to visit an institution or laboratory in another COST Member state. Their aim is to foster collaboration in excellent research infrastructures and share new techniques that might not be available in a participant’s home institution or laboratory. For example, an Early Career Investigator (ECI), from Albania will conduct research in Sweden and an ECI from Italy will be invited to engage in further studies in England.

A further step has been the design of a Training School in Porto, Portugal [4]. The European Research Workshop in Sexual Medicine aims to support ECI and PhD students in the fields of sexuality, sex research, sexual health, and sexual medicine by connecting them with experienced experts in the field. In Basel, Switzerland, a basic curriculum for university education on sexual medicine is being developed. The four WGs are well established and holding organisational meetings. One of them will prepare a communication and dissemination plan, as well as a communication strategy. Members are already planning specific actions to target the identified key players.

The Action’s website [1] constitutes a vital meeting point for stakeholders where they can post information about their research, initiate discussions and review new books and publications. A database will be freely available for stakeholders.

## The goals are ambitious

The ESMN overarching goal is to introduce and advance sexual medicine at every level of education, as well as in clinical and public health research and practice. The Action aims to exchange research results produced by different disciplines in order to find commonalities in concepts and approaches to sexual medicine. This will serve as the foundation for identifying shared concepts and definitions, and highlights the start of joint interdisciplinary research with a particular focus on including young researchers. It will also form the conceptual groundwork for developing interdisciplinary frameworks and curricula for further university education at a European standard of qualification and recognition.

The bar has been set high. The plan is that by the end of Year 1, the COST Research Network should have included participants from all major medical and health disciplines involved in sexual health and the treatment of sexual disorders. By the end of Year 2, the establishment of the COST Network will have stimulated new interdisciplinary research on sexual medicine, particularly conducted by ECI. The Network will have expanded by including less research-intensive COST Countries (Inclusiveness Target Countries), Near Neighbour Countries and International Partner Countries. Moreover, it will have expanded by including university policy makers, education specialists and representatives from patient organisations.

## Intended innovation

The ESMN advocates innovation by research and treatment in sexual medicine. It is a bottom-up initiative in the field of sexual medicine that closes the knowledge gap among medical, biological and psychosocial disciplines. It has the potential to overcome dated barriers between disciplines and also to consider sexual medicine as a holistic subject. ESMN concentrates on sharing knowledge, establishing a research agenda, developing joint research and creating an interdisciplinary approach.

One of the major challenges in sexual medicine is the daunting number of scientific arenas involved. The list is long and falls into four categories: research, collaboration, public services, standards and guidelines. The Network’s members are bringing innovative ideas to each category.

### Research: sexual medicine, psychology and social sciences

After consulting the secondary proposers the research subjects became apparent: effects of somatic disease and drugs on sexual functioning; iatrogenic sexual dysfunctions after medical interventions (radiotherapy, chemotherapy, etc.); infertility treatments; treatment alternatives for premature ejaculation; new directions for erectile dysfunction therapies; hypersexuality; healthy sexual aging; sexual health for young people including adolescents with special needs; LGBTQ health; the epidemiology of mental and sexual health disparities; structural HIV prevention concepts; syndemic science approaches; resilience; paraphilias; the role of sexualised media particularly online pornography; self-produced sexual images; prevention of online sexual offences; sexual health of vulnerable migrants; vulnerable groups and re-victimisation, and minority stress, and pharmaceutical products, such as sexually neutral antidepressants.

### Collaboration: exchange of information and research results among clinical and public health disciplines

The Network’s shared knowledge and know-how is now being applied to establish up-to-date curricula for university education that reflects current and anticipated future societal trends. Currently, in Europe curricula of this kind are only available to a limited extent. Being an international interdisciplinary body, the Action has the natural potential to stimulate innovations with immediate effect.

### Services: health services and advocacy

Network members will introduce health services, some of which can be tailored to mitigate stigma and facilitate health care access to promote the health of LGBTQ people. In addition, the members will design and manage thought-provoking websites focused on reproductive rights and sexual health advocacy including anti-sexual violence, safe-dating programs and parenthood. The times call for responsible programming in new media. Thus clips, videos and commentaries involving personal safety, self-help and the education of children will be created. For example, internet-based psychological interventions for special patient groups in order to cope with their disease, as well as for sexual minority adolescents.

### Standards and guidelines: significantly improve conditions for sexual health care

The Network sees itself as a standard bearer for the field no matter where its members are active—whether lecturing in distinguished institutions or in the public arena, in conducting far-reaching research or treating anxious individuals, in front of trailblazers or in innocent children’s classrooms. In sum, ESMN members and their work are leading the field with strength and conviction while advancing it wherever possible.

## Scientific, technological, and socioeconomic impacts

### Impact at a scientific level

The systematically organised exchange of well-developed, high-quality research which is undertaken and achieved on specific levels of discipline—thus connecting the disciplines’ “pockets of excellence” will lead to an increase in knowledge and understanding of the contributions that these disciplines may give to establish a robust and tangible basis for an all-inclusive approach to sexual medicine. The synthesis of research results which focus on identifying cross-discipline and cross-border commonalities will lead to shared concepts and definitions of sexual medicine. This is important in order to enable research and practice to remain up-to-date in relation to changes in society which have an impact on the subject matter, such as an ageing population and the increasing influence of (social) media on views and perceptions of sexual health.

Finally, the ESMN focus on providing the basis for a specific university curriculum on sexual medicine will reinforce the status of sexual medicine as a key input for diagnosis and later on for the treatment and care of patients with sexual disorders.

### Technological impact

Increased interdisciplinary knowledge and know-how could lead to specific patient-oriented technological innovations, e.g., as new pharmaceutical products and to the development and use of communication tools within the health care system and its different disciplines. From this perspective, innovations will contribute to an improvement in general diagnoses of sexual disorders that can be employed by specific clinical disciplines.

### Socioeconomic impact

Sexual health covers many areas of sexuality, i.e., sexual satisfaction at all stages of life, the sexual health of adolescents, unwanted pregnancies and safe abortions, sexual dysfunctions, reproductive health, sexual violence, HIV/STD, the influence of chronic diseases and handicaps, sexual orientation, sexual identity, psychological health, the support of safe, sensual sexual experiences and psychological health.

The Action will have a significant socioeconomic influence on heterogeneous political frameworks and social determinants such as strategies, concepts, legal regulations, guidelines and on an improved access to family planning, reproductive health, STD-diagnostics and therapy, sex education and care for victims of sexual violence. The benefit for the health care system will be enormous.

Due to different cultures, traditions, doctrines and established expectations within Europe and abroad, women’s choice for birth control is to a greater or lesser extent limited within the Network countries. Depending on the region, there is still an unmet need for contraception. The ESMN will stand for enhanced public education about birth control methods and also for access to birth control.

The socio-economic impact can be identified at different levels. Firstly, identifying commonalities in concepts and definitions will lead to a more robust and shared understanding of the position of sexual medicine in medical science. Secondly, this will impact the education of physicians who will be better equipped to identify sexual disorders. There will be a new pool of competent researchers: this will also have a positive effect on clinical practice.

The ESMN is an excellence-driven and inclusive Network for peaceful purposes in all areas of sexual medical science and technology. The doors are open to get in touch with the ESMN.
